# Supplementary material for: The Single and Combined Effects of Prenatal Nonchemical Stressors and Lead Exposure on Neurodevelopmental Outcomes in Toddlers: Results from the CCREOH Environmental Epidemiologic Study in Suriname
Source: Children (Basel). 2023 Feb 2;10(2):287. doi: 10.3390/children10020287 (PMC9954975; doi:10.3390/children10020287)
Supplement: Supplementary file 1 [file children-10-00287-s001.zip › children-2089662-supplementary.pdf]

Supplementary Materials

Table S1. Comparison of participants with and without BSID III assessments (n = 666).

Variables	**with BSID III assessment (n = 363)**	**without BSID III assessment (n = 303)**	*p-value*
Blood Lead			
Median (IQR)	1.74 (1.13–2.57)	1.96 (1.31–2.75)	0.103
Blood lead level ≥ 3.5 μg/dl	11.1%	16.1%	0.239
Blood lead level < 3.5 μg/dl	88.9%	83.9%	
Cohen's Perceived Stress Scores			
Median (IQR)	16 (12–19)	18 (14–20)	0.004
20–40 high	24.6%	34.9%	0.004
0–19 low–normal	75.4%	65.1%	
Edinburgh Depression Scores			
Median (IQR)	7 (4–10)	8 (5–12)	0.038
0–6 no or minimal	46.2%	41.4%	0.335
7–13 mild	41.8%	42.5%	
14–19 moderate	8.8%	13.0%	
20–30 severe	3.2%	3.1%	
Age			
Median (IQR)	28.4 (24.7–33.0)	27.6 (23.3–32.6)	0.228
16–19	8.8%	11.6%	0.074
20–24	17.9%	23.8%	
25–29	32.2%	23.4%	
30–34	22.6%	22.8%	
35+	18.5%	18.5%	
Parity			
0–3 previous live births	90.9%	90.4%	0.819
4+ previous live births	9.1%	9.6%	
Ethnic background			
Creole	34.4%	32.7%	0.778
Hindustani	17.6%	17.5%	
Indigenous	1.4%	1.0%	
Javanese	3.9%	5.3%	
Tribal	18.7%	22.1%	
Mixed	24.0%	21.5%	
Educational level			
primary or not educated	10.2%	13.5%	0.157
lower secondary/vocational	34.2%	38.9%	
upper secondary/vocational	33.1%	30.0%	
tertiary	22.6%	17.5%	
Household income in SRD			
< 800	6.6%	7.0%	0.089
800–1499	17.3%	17.6%	
1500–2999	32.7%	41.2%	
3000+	43.4%	34.2%	
Household size			

< 3 persons	10.7%	13.6%	0.257
3+ persons	89.3%	86.4%	
Marital status			
Married/living together	81.3%	81.7%	0.879
Unmarried/single	18.7%	18.3%	

Table S2. Comparison of participants with BSID III data with and without blood lead levels (n = 363).

Variables	**BSID III with blood lead level (n = 153)**	**BSID III without blood lead level (n = 210)**	***p*-value**
Cohen's Perceived Stress Scores			
Median (IQR)	17 (13–19)	16 (12–20)	0.631
20–40 high	23.0%	25.8%	0.556
0–19 low–normal	77.0%	74.2%	
Edinburgh Depression Scores			
Median (IQR)	7 (4–10)	7.0 (4–10)	0.716
0–6 no or minimal	45.9%	46.4%	0.143
7–13 mild	43.9%	40.1%	
14–19 moderate	7.4%	9.9%	
20–30 severe	2.7%	3.6%	
Age			
Median (IQR)	28.5 (24.6-34.2)	28.4 (24.6-32.5)	0.578
16–19	8.5%	9.0%	0.937
20–24	17.0%	18.6%	
25–29	31.4%	32.9%	
30–34	24.8%	21.0%	
35+	18.3%	18.6%	
Parity			
0–3 previous live births	90.8%	90.9%	0.985
4+ previous live births	9.2%	9.1%	
Ethnic background			
Creole	33.7%	35.7%	0.111
Hindustani	19.0%	16.7%	
Indigenous	0.0%	2.4%	
Javanese	3.3%	4.3%	
Tribal	15.7%	21.0%	
Mixed	29.4%	20.0%	
Educational level			
primary or not educated	7.2%	12.4%	0.075
lower secondary/vocational	41.2%	29.0%	
upper secondary/vocational	30.7%	34.8%	
tertiary	20.9%	23.8%	
Household income in SRD			
< 800	9.2%	4.9%	0.047
800–1499	18.4%	16.6%	
1500–2999	24.8%	38.0%	
3000+	47.5%	40.5%	
Household size			
< 3 persons	9.8%	11.4%	0.622
3+ persons	90.2%	88.6%	
Marital status			
Married/living together	80.4%	81.9%	0.715
Unmarried/single	19.6%	18.1%	

Table S3. Correlations (*r* (95% CI)) between maternal NCSs, lead levels, and demographics.

	Age	Parity	Ethnic background	Educational level	Household size	Household income	Marital status	Stress	Probable depression	Lead levels
Age		0.02 (0.00–0.62)	0.01 (0.00–0.19)	0.00 (0.00–0.22)	0.00 (0.00–0.21)	0.00 (0.00–0.24)	0.00 (-0.06–0.00)	0.00 (0.00–0.30)	0.05 (0.00–0.01)	0.00 (0.00–0.14)
Parity	0.02 (0.00–0.62)		0.00 (0.00–0.36)	0.00 (-0.38–0.00)	0.00 (0.00–0.65)	0.00 (-0.08–0.00)	0.00 (0.00–0.41)	0.00 (0.00–0.10)	0.03 (0.00–0.31)	0.00 (-0.17–0.00)
Ethnic background	0.01 (0.00–0.19)	0.00 (0.00–0.36)		0.00 (0.00–0.11)	0.00 (0.00–0.22)	0.01 (0.00–0.17)	0.00 (0.00–0.05)	0.00 (0.00–0.18)	0.04 (0.00–0.15)	0.00 (0.00–0.53)
Educational level	0.00 (0.00–0.22)	0.00 (-0.38–0.00)	0.00 (0.00–0.11)		0.00 (-0.03–0.00)	0.00 (-0.03–0.00)	0.05 (-0.15–0.02)	0.00 (0.00–0.47)	0.06 (-0.18–0.00)	0.00 (0.00–0.56)
Household size	0.00 (0.00–0.21)	0.00 (0.00–0.65)	0.00 (0.00–0.22)	0.00 (-0.03–0.00)		0.00 (0.00–012)	0.00 (0.00–0.40)	0.00 (0.00–0.27)	0.00 (0.00–0.23)	0.00 (0.00–033)
Household income	0.00 (0.00-0.24)	0.00 (-0.08–0.00)	0.01 (0.00–0.17)	0.05 (0.16–0.69)	0.00 (0.00–012)		0.03 (-0.12–0.00)	0.00 (0.00–0.30)	0.05 (-0.12–0.00)	0.00 (0.00–037)
Marital status	0.00 (-0.06–0.00)	0.00 (0.00–0.41)	0.00 (0.00–0.05)	0.05 (-0.15–0.02)	0.00 (0.00–0.40)	0.03 (-0.12–0.00)		0.00 (-0.05–0.00)	0.04 (0.00–0.43)	0.00 (0.00–0.29)
Stress	0.00 (0.00–0.30)	0.00 (0.00–0.10)	0.00 (0.00–0.18)	0.00 (0.00–0.47)	0.00 (0.00–0.27)	0.00 (0.00–0.30)	0.00 (-0.05–0.00)		0.04 (-0.44–0.08)	0.00 (0.00–0.37)
Probable depression	0.05 (0.00–0.01)	0.03 (0.00–0.31)	0.04 (0.00–0.15)	0.06 (-0.18–0.00)	0.00 (0.00–0.23)	0.05 (-0.12–0.00)	0.04 (0.00–0.43)	0.07 (-0.44–0.00)		0.00 (0.00–0.07)
Lead levels	0.00 (0.00–0.14)	0.00 (-0.17–0.00)	0.00 (0.00–0.53)	0.00 (0.00–0.56)	0.00 (0.00–033)	0.00 (0.00–037)	0.00 (0.00–0.29)	0.00 (0.00–0.37)	0.00 (0.00–0.07)	
